# Supplementary material for: Congenital Heart Disease: Growth Evaluation and Sport Activity in a Paediatric Population
Source: Children (Basel). 2022 Jun 14;9(6):884. doi: 10.3390/children9060884 (PMC9221605; doi:10.3390/children9060884)
Supplement: Supplementary file 1 [file children-09-00884-s001.zip › children-1739839-supplementary.pdf]

Supplementary Material

Table S1. Patients with comorbidities excluded to the analysis.

**COMORBIDITY**	**N**	**%**
Isolated patent oral foramen	32	45.71
Down syndrome	10	14.29
Unspecific conditions causing	4	5.71
Marfan Syndrome	2	2.86
Autism	2	2.86
Noonan syndrome	2	2.86
Di George syndrome	2	2.86
Turner syndrome	2	2.86
Scoliosis and Hemiparesis	1	1.43
Oesophagus Atresia	1	1.43
Patient on growth hormone therapy	1	1.43
Jacobsen syndrome	1	1.43
Chromosome 6 deletion	1	1.43
Charge syndrome	1	1.43
Saethre Chtozen syndrome	1	1.43
Ellis Van Creveld syndrome	1	1.43
Kabuki syndrome	1	1.43
Chromosome 12p and 10q deletion	1	1.43
Chromosome 4 deletion	1	1.43
Thrisomy chromosome x	1	1.43
Chromosome 22 deletion	1	1.43
Syringomyelia	1	1.43
	70	100
